# Activity of growth factors in the IL-6 group in the differentiation of human lung adenocarcinoma

**DOI:** 10.1054/bjoc.1999.1015

**Published:** 2000-02

**Authors:** C McCormick, R I Freshney

**Affiliations:** CRC Department of Medical Oncology, University of Glasgow, Alexander Stone Building, Garscube Estate, Bearsden, Glasgow, G61 1BD, UK

**Keywords:** differentiation, interleukin-6, oncostatin M, NSCLC, alkaline phosphatase, SPA

## Abstract

The role of the interleukin-6 (IL-6) group of cytokines in differentiation of two lung adenocarcinoma cell lines has been examined using induction of alkaline phosphatase and expression of surfactant protein A. Oncostatin M was the most active and potent for alkaline phosphatase in A549 cells, with IL-6 having similar activity but less potency. Neither cytokine induced alkaline phosphatase in NCI-H441 cells, although induction was obtained with lung fibroblast-conditioned medium. Surfactant protein A was induced in NCI-H441 cells by conditioned medium and dexamethasone and, to a much lesser extent, by oncostatin M or IL-6. Induction of alkaline phosphatase and surfactant protein A were both dexamethasone-dependent, though some induction of surfactant protein A was obtained with interferon-α in the absence of dexamethasone. The activity present in lung fibroblast-conditioned medium suggests paracrine control, but this appears not to be due to oncostatin M or IL-6 as disabling antibodies to either cytokine were not inhibitory, and, although alkaline phosphatase was induced in A549 by both cytokines, it was only induced by conditioned medium in NCI-H441 cells. Furthermore, surfactant protein A was induced in H441 by conditioned medium to a much greater extent than by oncostatin M or IL-6. These data demonstrate that cytokines of the IL-6 group have potential as differentiation inducers in lung adenocarcinoma cells and that there is an equivalent paracrine factor(s) in lung fibroblast conditioned medium. As the production of this factor by fibroblasts is not enhanced by glucocorticoid, although the response of the target cell is, it would appear to be distinct from the fibrocyte pneumocyte factor previously described by Post et al 1984 *Nature***308**: 284–286. © 2000 Cancer Research Campaign


British Journal of Cancer (2000) **82**(4), 881–890
© 2000 Cancer Research Campaign
DOI: 10.1054/ bjoc.1999.1015, available online at http://www.idealibrary.com on IDEAL®

### Activity of growth factors in the IL-6 group in the differentiation of human lung adenocarcinoma

**C McCormick and RI Freshney**

CRC Department of Medical Oncology, University of Glasgow, Alexander Stone Building, Garscube Estate, Bearsden, Glasgow G61 1BD, UK

**Summary** The role of the interleukin-6 (IL-6) group of cytokines in differentiation of two lung adenocarcinoma cell lines has been examined using induction of alkaline phosphatase and expression of surfactant protein A. Oncostatin M was the most active and potent for alkaline phosphatase in A549 cells, with IL-6 having similar activity but less potency. Neither cytokine induced alkaline phosphatase in NCI-H441 cells, although induction was obtained with lung fibroblast-conditioned medium. Surfactant protein A was induced in NCI-H441 cells by conditioned medium and dexamethasone and, to a much lesser extent, by oncostatin M or IL-6. Induction of alkaline phosphatase and surfactant protein A were both dexamethasone-dependent, though some induction of surfactant protein A was obtained with interferon-α in the absence of dexamethasone. The activity present in lung fibroblast-conditioned medium suggests paracrine control, but this appears not to be due to oncostatin M or IL-6 as disabling antibodies to either cytokine were not inhibitory, and, although alkaline phosphatase was induced in A549 by both cytokines, it was only induced by conditioned medium in NCI-H441 cells. Furthermore, surfactant protein A was induced in H441 by conditioned medium to a much greater extent than by oncostatin M or IL-6. These data demonstrate that cytokines of the IL-6 group have potential as differentiation inducers in lung adenocarcinoma cells and that there is an equivalent paracrine factor(s) in lung fibroblast conditioned medium. As the production of this factor by fibroblasts is not enhanced by glucocorticoid, although the response of the target cell is, it would appear to be distinct from the fibrocyte pneumocyte factor previously described by Post et al 1984 *Nature* **308**: 284–286. © 2000 Cancer Research Campaign

**Keywords:** differentiation; interleukin-6; oncostatin M; NSCLC; alkaline phosphatase; SPA

Paracrine regulation of differentiation has been demonstrated to be implicated in the induction and maintenance of differentiation in several different developmental systems. Perhaps the best documented, and now classical, model, is the induction of growth and differentiation in prostatic epithelium where stimulation by testosterone requires the presence of functional receptors in the stromal component of the gland (Cunha et al, 1983). Treatment of stromal fibroblasts induces the release of keratinocyte growth factor (KGF, FGF-7) which binds to a specific receptor of the FGFR family, *Bek*, only present on the epithelium, and elicits proliferation and maturation of the epithelium (Yan et al, 1992; Planz et al, 1998). Similar indirect hormonal control of differentiation has been demonstrated in the intestine (Kedinger et al, 1987), where the action of hydrocortisone requires the presence of stroma in order to induce differentiation in the enterocytes, and in the lung, where the perinatal production of surfactant lipid by type II pneumocytes is regulated by hydrocortisone via stimulation of lung fibroblasts (Post et al, 1984; Nielsen et al, 1992). While direct cell contact, or indirect cell–cell interaction via constituents of the extracellular matrix, may be implicated in some of these interactions (Simon-Assmann, 1986), there is also a significant role for diffusible factors, such as KGF in the prostate (Yan et al, 1992). In the lung, a factor has been described, called fibrocyte-pneumocyte factor (FPF) (Post et al, 1984), detectable in conditioned medium from primary cultures of lung fibroblasts, but so far, this has not been purified to homogeneity, cloned, or fully characterized. Differentiating activity is present in fibroblast-conditioned medium (CM) from cell lines (McCormick et al, 1995), but not restricted to lung fibroblasts.

The prospect of inhibiting tumour growth by induction of differentiation has received considerable attention and has given promising results in vitro and in vivo. However, the use of agents such as the planar-polar compound hexamethylene bisacetimide (HMBA) (Marks et al, 1996), has had limited clinical success due to the difficulty in maintaining the high concentration required and ensuing problems of toxicity. More promising results have been obtained using retinoids, where enhanced response rates have been obtained in acute promyelocytic leukaemia (Drach et al, 1993) and potentiation with HMBA is possible (Taimi et al, 1998). This suggests that other natural high potency regulators of differentiation, such as growth factors or cytokines implicated in paracrine control of differentiation, may be worthy of further exploration.

Speirs et al (1991) showed that a paracrine factor from lung fibroblasts treated with dexamethasone (DX, a synthetic analogue of hydrocortisone), was able to induce differentiation (induction of pulmonary surfactant synthesis), in the A549 cell, a tumour of type II pneumocyte or Clara cell origin. McCormick et al (1995), in a screen of potential candidates for this paracrine activity, showed that insulin, interferon-α (IFN-α) and interleukin (IL)-6 are all active inducers of differentiation, by demonstrating induction of alkaline phosphatase (AP), another marker of type II cell differentiation. As IL-6 was one of the most active, we have now examined other growth factors in the same family. We have also used blocking antibodies to determine whether any of these are present, and active, in fibroblast-CM.

*Received 16 September 1998*
*Revised 5 October 1999*
*Accepted 19 October 1999*

*Correspondence to:* RI Freshney

IL-6 is one of a group of growth factors operating through the Gp130 receptor sub-unit (Taga and Kishimoto, 1997). Others in the group include IL-11, ciliary neurotropic factor (CNTF), oncostatin-M (OSM) and leukaemia inhibitory factory (LIF). Granulocyte-colony stimulating factor (G-CSF) has been included in this group (Rose and Bruce, 1991; Bruce et al, 1992), and also acts via the JAK-STAT pathway (Akira, 1997) but most recent reviews exclude it (e.g. Taga and Hishimoto, 1997). G-CSF, LIF and IL-11 are differentiation and proliferation factors, active principally in the haematopoietic system (Taga et al, 1992; Katayama and Ogawa, 1994), and CNTF in the neural system (Lillien et al, 1990). LIF is an inhibitor of differentiation of embryonal stem (ES) cells (Boeuf et al, 1997; Niwa et al, 1998), and the other members of the group have similar, though less potent, activity on ES cell differentiation (Bruce et al, 1992).

In the present report we have confirmed the activity of IL-6 and shown that OSM is significantly more active and potent than IL-6 in the induction of A549 differentiation and, like all other factors previously investigated, with the exception of FGF-2, is dependent on the presence of DX for its activity. LIF and IL-11 are also active, though less so than OSM, and CNTF and G-CSF are inactive. Transforming growth factor (TGF)-β is inhibitory in this system (McCormick et al, 1995) and we now show that it blocks the inductive activities of OSM and IL-6.

The potential roles of IL-6 and OSM as paracrine factors produced by lung fibroblasts were tested by use of disabling antibodies. The results failed to show any reduction in the inductive effect of CM, in spite of demonstrable inhibition of induction of AP by recombinant human OSM (rhOSM) and rhIL-6. Furthermore, DX and CM induced AP and SPA in NCI-H441 cells, another Type II/Clara cell-derived tumour cell line, while OSM and IL-6 induced SPA but not AP.

### MATERIALS AND METHODS

### Cell culture

A549 (CCL-185) and NCI-H441 (HTB-174) cells were obtained from the American Tissue Culture Collection (ATCC) and maintained in a 50:50 mixture of Dulbecco's modification of Eagle's medium and Ham's F10 (F10:DMEM) (Life Technologies), supplemented with 10% fetal bovine serum (FBS; Globepharm). MOG-LF113 cells, used as a source of conditioned medium, are normal human diploid lung fibroblasts derived from a first trimester embryo. They were used between generations 10 and 30. Cell lines were tested monthly for mycoplasma by fluorescence staining with Hoechst 33258 (Chen, 1977).

### Conditioned medium

Fibroblast CM was prepared from LF113 cells, seeded at $2 \times 10^4$ cells $ml^{-1}$, grown to confluence, and grown on for 8 days in F10:DMEM with 0.5% FBS. To prepare CM, the medium was replaced with serum-free F10:DMEM, or, to prepare DXCM, with serum-free F10:DMEM containing 0.25 μM DX, for a further 3 days, collected and stored frozen. CM was thawed, filtered through a 0.22 μm Millipore GV filter, and used at 50% (unless otherwise indicated) in fresh serum-free F10:DMEM.

### Alkaline phosphatase activity

Cell monolayers in microtitration plates were washed in 0.15 M sodium chloride (NaCl) and frozen and thawed $3 \times$ in 10 μl 0.15 M NaCl. AP was assayed by the dephosphorylation of *p*-nitrophenyl phosphate at pH 8.5 (Sigma kit #104). The concentration of coloured product, *p*-nitrophenol (PNP), was determined by absorbance at 310 nm, with reference to a standard curve. Enzyme activity is expressed as μmol PNP produced $h^{-1}$ $10^{-5}$ cells (counted in replicate wells). The reaction was shown to be linear over the range of activities measured (data not shown).

### Growth factors, antibodies and other reagents

IL-6 was obtained from Boehringer Mannheim, OSM from NBS Biologicals, LIF and IL-11 from Genetics Institute, G-CSF from Amgen, CNTF from Pepro Tech, TGF-β from British Biotechnology, anti-IL-6 antibody from NIBSC and anti-OSM from R&D. DX was obtained from Sigma (#D1756) and used at 0.25 μM, unless indicated otherwise. Actinomycin D (#A1410) and cycloheximide (#C6255) were obtained from Sigma and used at 5.0 μg $ml^{-1}$, at which concentration no loss of viability was observed using naphthalene black as a viability stain (Freshney, 2000).

### Assay for exposure to growth factors and CM

A549 cells were seeded at $5 \times 10^4$ cells $ml^{-1}$, grown for 3 days and the medium changed to serum-free F10:DMEM. Growth factors or CM were added for a further 3 days and the AP activity measured as above.

### Exposure to antibodies

CM or growth factors were exposed to antibodies at a range of concentrations, including zero, in polypropylene microtubes (Eppendorf) for 2 h at 37°C, immediately before addition to assay plates, and the assay continued as above.

### Northern blots

Northern blots were prepared by standard procedures (Sambrook et al, 1989). Blots were probed with Probes 1255 and 2305, obtained from Riken.

### RESULTS

Incubation of A549 cells for 3 days with DX alone, in the absence of serum, induced AP three-fold at 0.25 μM ($P < 0.0001$) and 4.8-fold at 10 μM ($P < 0.0001$), but not at all at 50 nM (Figure 1). IL-6 added for 3 days in the presence of 0.25 μM DX, gave a dose-dependent increase in AP activity, reaching a maximum at 2.5 μg $ml^{-1}$, amounting to $2.3 \times$ DX alone in 0.25 μM ($P < 0.0001$), and $2 \times$ DX alone in 10 μM ($P < 0.0001$).

OSM, under similar conditions, induced AP 2.6-fold at 20 ng $ml^{-1}$ ($P = 0.0036$), and was, therefore, both more potent and more active than IL-6 (Figure 2). Like IL-6, it required DX for activity; it was inhibitory on its own. LIF gave 50% induction at 200 U $ml^{-1}$ ($P < 0.0001$) and IL-11 60% induction at 1000 U $ml^{-1}$ ($P = 0.0001$), in the presence of 0.25 μM DX (Figure 3), and were inactive in the absence of DX. CNTF and G-CSF gave no

© 2000 Cancer Research Campaign

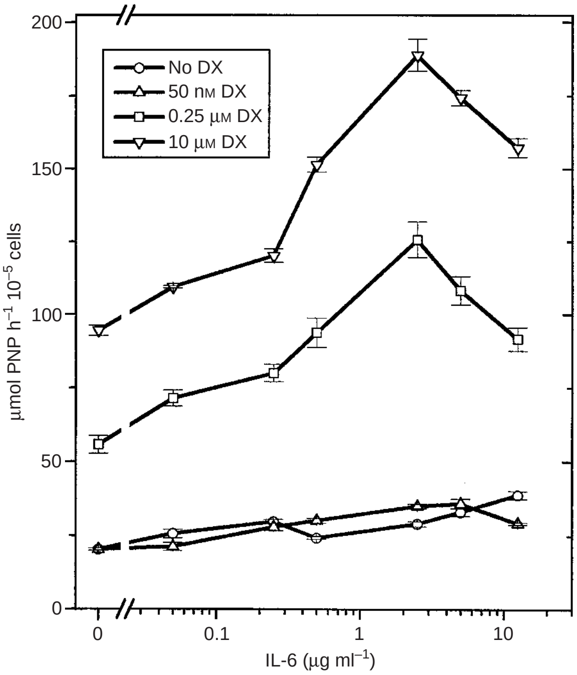

**Figure 1** Effect of DX concentration on induction of AP by IL-6 in A549 cells. Mid-log phase A549 cells were incubated in microtitration plates in serum-free medium for 3 days in DX at three different concentrations and a range of concentrations of IL-6 from 0.5 to 12.5 μg ml$^{-1}$, and assayed for AP as described in the Methods. Means ± s.e.m.

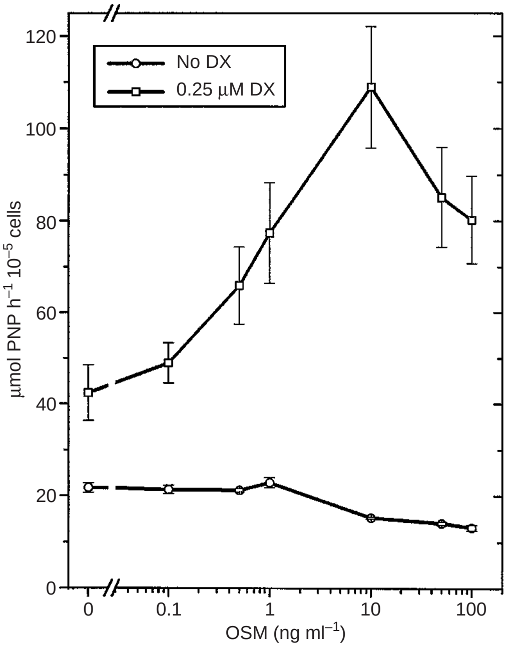

**Figure 2** Effect of DX on induction of AP by OSM in A549 cells. Mid-log phase A549 cells were incubated in microtitration plates in serum-free medium for 3 days with or without DX and a range of concentrations of OSM from 0.1 to 100 ng ml$^{-1}$, and assayed for AP as described in the Methods. Means ± s.e.m significant induction ($P$ > 0.05). The combination of optimal concentrations of IL-6 and OSM showed about 15% more activity than either IL-6 or OSM alone but was not additive (data not shown).

TGF-β was shown previously to be inhibitory for the induction of AP in A549 cells (McCormick et al, 1995) and was able to obliterate the effects of either IL-6 or OSM in the present series (Figure 4).

As previously reported (McCormick et al, 1995), CM from a lung fibroblast cell line, LF113, induced AP activity in the presence of DX. Unlike the induction of pulmonary surfactant (Speirs et al, 1991), prior exposure of the fibroblasts to DX did not increase the AP-inductive capacity of CM, and, in fact, reduced its potency, perhaps due to down regulation of IL-6 (McCormick et al, 1995) (Figure 5). The induction by CM was completely blocked by 5.0 μg ml$^{-1}$ cycloheximide and 5.0 μg ml$^{-1}$ actinomycin D (Figure 6).

In order to determine whether IL-6 or OSM were potential candidates for paracrine differentiation factors present in CM, CM was treated with disabling antibodies to IL-6 and OSM. While both antibodies inhibited their respective cytokines in positive controls (Figure 7 B, D), no inhibition of activity in CM was observed (Figure 7 A, C).

AP activity is used in these experiments as a marker of differentiation. To confirm that differentiation was being induced, expression of mRNA for SPA was determined in A549 and NCI-H441 cells. There was little induction of SPA by DX alone in A549. CM+DX, DXCM, OSM and IL-6 all showed less SPA expression than the serum-free control (SF) or DX alone, although the DX values were always higher than CM or cytokines alone. In H441 cells, SPA and AP were both induced by DX and CM (Figures 8 and 9). DX ($P$ = 0.02), CM+DX ($P$ = 0.0003) and DXCM ($P$ = 0.003) induced AP, with greatest activity in CM+DX, but OSM and IL-6 showed no AP induction over DX alone. IFN-α, previously shown to induce AP in A549 cells (McCormick et al, 1995), also induced AP in H441 ($P$ = 0.06). Preliminary data from Northern analysis suggest that CM+DX and DXCM induce SPA expression in H441, while OSM, IL-6, insulin and interferon, in the presence of DX, also induced SPA relative to DX alone (Figure 9), although expression was never as great as with CM+DX.

### DISCUSSION

Previous results indicated that IL-6 was an active inducer of AP in A549 cells in the presence of DX (McCormick et al, 1995), prompting the investigation of other members of this family of cytokines. Among those studied, the highest activity and greatest potency were found with OSM, with 2.6-fold stimulation at 10 ng ml$^{-1}$, compared with 2.3-fold stimulation with IL-6 at 2.5 μg ml$^{-1}$. Activity was also seen with LIF (50% at 200 U ml$^{-1}$) and IL-11 (60% at 1000 U ml$^{-1}$). The response to IL-11 may have been cross-reaction with one of the other receptors, as the concentration of ligand was quite high, but the response to LIF, although modest, may have been through the LIF receptor, known to be expressed on A549 (Piquet-Pellorce et al, 1994). TGF-β stimulates A549 cells to produce IL-11 and DX inhibits this stimulation (Wang et al, 1999) so, even if IL-11 receptors are present on A549 the stimulatory role of TGF-β and inhibitory role of DX would conflict with the effects seen here with AP induction. No significant activity was found with G-CSF and CNTF. Activities may be ranked as follows: OSM > IL-6 > LIF > IL-11 > G-CSF > CNTF.

This group of cytokines has been shown to share activities in other systems, e.g. inhibition of differentiation in ES cells, where

*© 2000 Cancer Research Campaign*

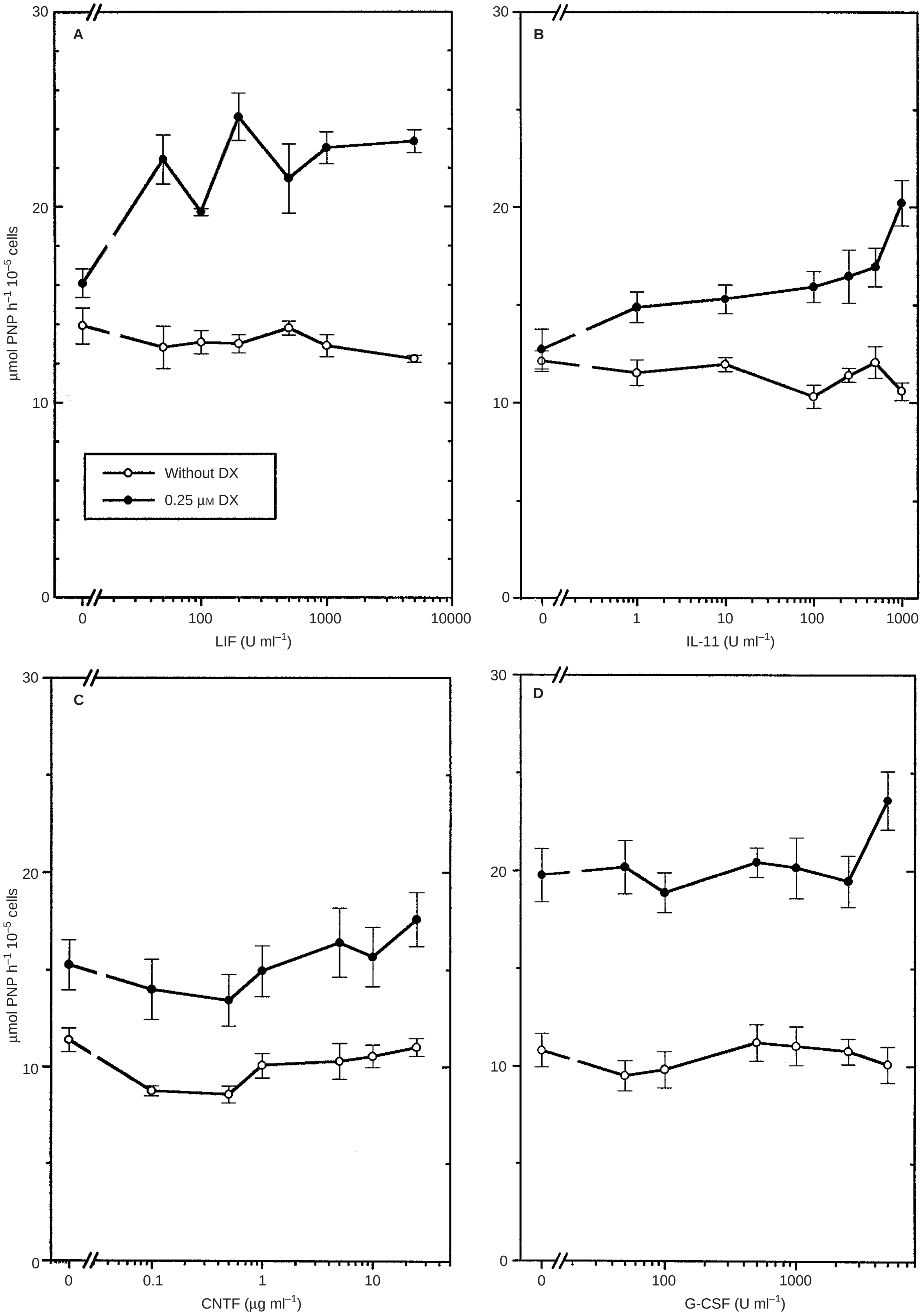

**Figure 3** Induction of AP by other members of the IL-6 family. Mid-log phase A549 cells were incubated in microtitration plates in serum-free medium for 3 days with or without DX and a range of concentrations of LIF, IL-11, CNTF, and G-CSF, and assayed for AP as described in the Methods. Means ± s.e.m

 *© 2000 Cancer Research Campaign*

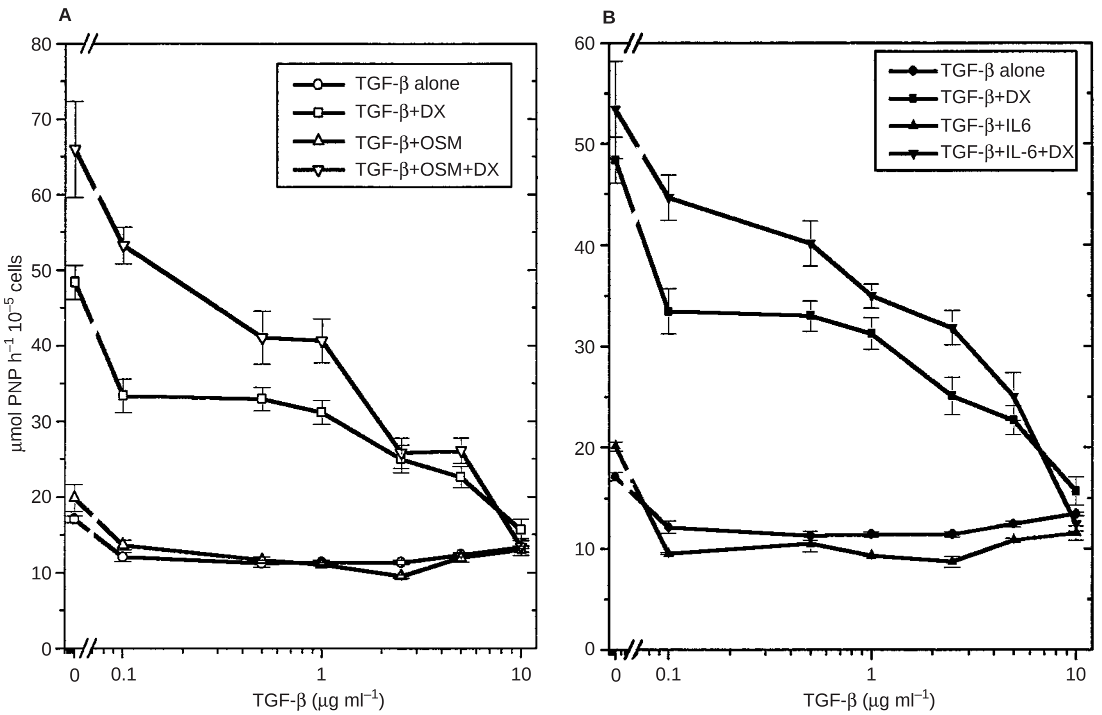

**Figure 4** Effect of TGF-β on induction of AP by OSM. Mid-log phase A549 cells were incubated in microtitration plates in serum-free medium for 3 days with or without DX and OSM (**A**) or IL-6 (**b**) and a range of concentrations of TGF-β, and assayed for AP as described in the Methods. Means ± s.e.m

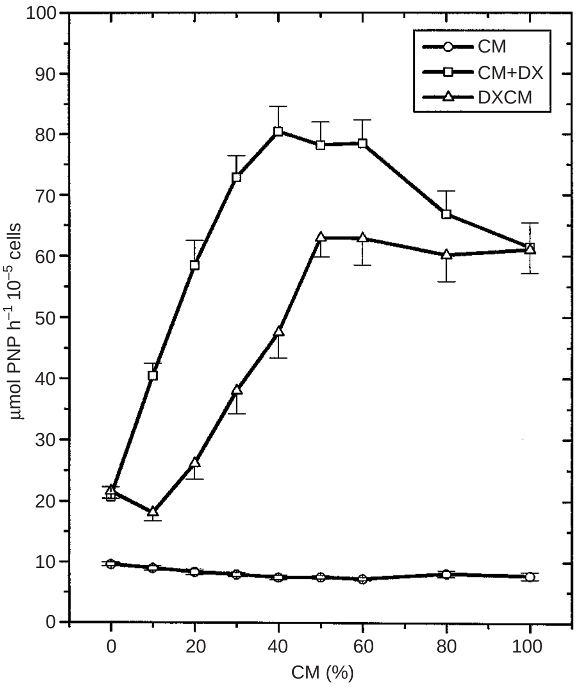

**Figure 5** Induction of AP in lung fibroblast-conditioned medium (CM). CM was prepared as described in the Methods and diluted in serum-free F10:DMEM in the percentages shown. Mid-log phase A549 cells were incubated in microtitration plates in serum-free medium for 3 days with different concentrations of condition medium and assayed for AP as described in the Methods. CM is fibroblast-conditionED medium diluted in F10:DMEM; CM+DX is CM with 0.25 µM DX added; DXCM is CM prepared from lung fibroblasts, LF113, in the presence of 0.25 µM DX. Means ± s.e.m

LIF has the greatest effect (Smith et al, 1988). It has been suggested that the shared activity results from a common interaction with the Gp130 subunit of the receptor, which they all share, while specificity is determined by the α-subunit which is unique to each cytokine (Gearing et al, 1992; Taga and Kishimoto, 1997). Low concentrations of the active cytokines are additive, while high concentrations are not (data not shown), again suggesting a shared receptor.

Activity was only seen in the presence of DX at a minimum concentration of 0.25 µM. The effect of DX is likely to be multifactorial. Glucocorticoids are known to influence gene transcription when the nuclear receptor binds to the glucocorticoid response element (GRE) (Reichardt and Schutz, 1998) and modulate genes activated by OSM (Haselmann and Goppelt-Struebe, 1997). In addition, they can influence signal transduction by interaction with the IL-6 signal transduction pathway, JAK tyrosine kinase and the STAT transcriptional activator (Takeda et al, 1998), and by inhibition of protein kinase C (PKC) action downstream of PKC activation (Tian et al, 1999). They can also affect cytokine receptor presentation (Wiegers and Reul, 1998), inhibit extracellular proteases (Freshney, 1985) which, in turn, can block activation of TGF-β (Cai et al, 1997).

DX has also been shown to alter the extracellular matrix (Mackie et al, 1988) and extracellular matrix has been shown to be important in promoting the expression of the type II phenotype in culture (Liu and Mautone, 1996; Alcorn et al, 1997). In the current system DX has been found to increase the synthesis of a fraction of heparan sulphate present in the medium of DX-treated A549 cells. This fraction is heparinase and heparitinase-sensitive and can replace DX in the induction of AP in A549 cells by CM, IL-6 and OSM (Yevdokimova and Freshney, 1997). CNTF, a member of the IL-6 family, has been shown to interact with matrix in the differentiation of type 2 astrocytes (Lillien et al, 1990), and heparan

© 2000 Cancer Research Campaign

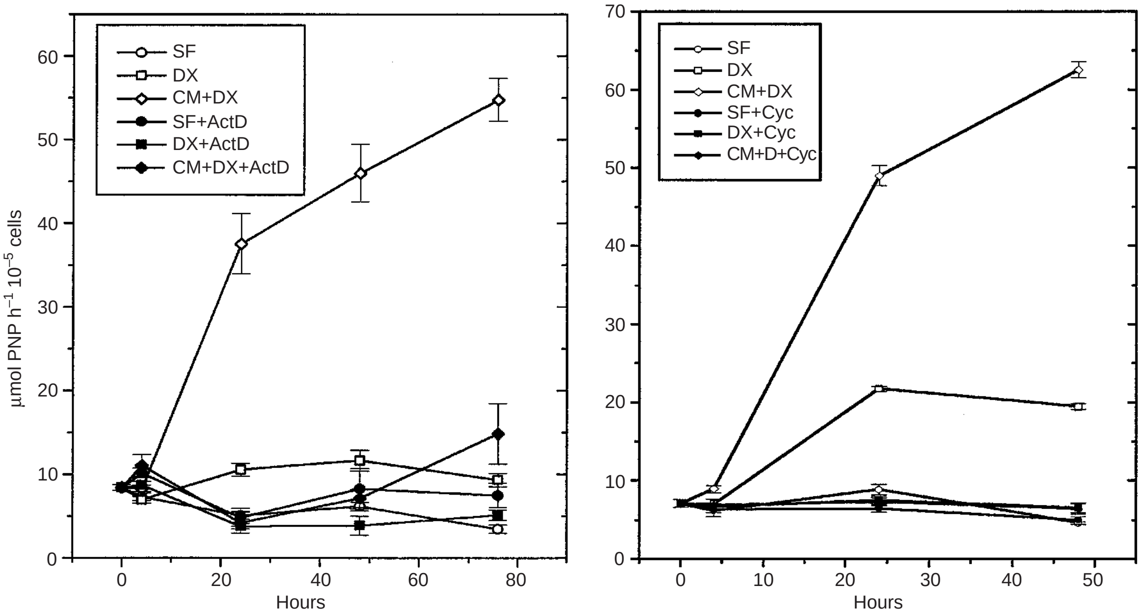

**Figure 6** Effect of actinomycin D and cycloheximide on induction of AP. Mid-log phase A549 cells were incubated in microtitration plates in serum-free medium for the time shown, with or without CM, in the presence or absence of 0.25 µM DX, and with or without 5 µg ml$^{-1}$ actinomycin D (ActD) or cycloheximide (Cyc) and assayed for AP as described in the Methods. Means ± s.e.m sulphate proteoglycan (HSPG) has been implicated in receptor dimerization (Schlessinger et al, 1995). It would appear that, if induction of an HSPG is the main role of DX in this system, then either the HSPG shares affinity for a wide range of growth factors, including IF-α, IF-β, OSM, IL-6 and insulin, all of which have been shown to have DX-dependent activity (McCormick et al, 1995, and this report), or they all induce a secondary autocrine factor to be produced by the target cells, A549 or H441, and the specificity of the HSPG is directed towards that factor.

The activities of both OSM and IL-6 were inhibited by TGF-β, consistent with the inhibitory role proposed for TGF-β in type II cell maturation (Torday and Kourembanas, 1990; Beers et al, 1998), where TGF-β in the lung remains high during embryonic development but falls just before parturition when hydrocortisone levels rise, inhibit TGF-β3 gene expression and induce surfactant production (Jaskoll et al, 1996). Although TGF-β is low at parturition, it can be induced by hydrocortisone in embryonic rat lung fibroblasts and may be implicated in the morphogenesis of bronchial epithelial cells (Wang et al, 1995). Preparation of fibroblast-conditioned medium in the presence of DX decreased its inductive effect on A549 cells (see Figure 5) which would be compatible with induction of TGF-β in the fibroblasts. The effect of TGF-β on SPA expression was not investigated in this study, but inhibition in H441 has been reported (Whitsett et al, 1992).

As IL-6 and OSM are both potential candidates for the inducing activity of CM, blocking antibodies were used to determine whether they could account for all or part of the activity of CM. Antibodies to IL-6 and OSM, selected for their ability to inhibit the pure cytokine, were unable to inhibit CM. This suggests that the major active component of CM is neither IL-6 nor OSM, although it is acknowledged that native IL-6 or OSM in conditioned medium may be in a different form from the recombinant cytokines to which the antibodies were raised, e.g. bound to a cofactor such as a proteoglycan (Yevdokimova and Freshney, 1997) or by post-translational modification. However, CM, in the presence of DX, induced both AP activity and SPA gene expression in H441 cells, while OSM and IL-6 induced SPA but not AP, although some AP-inducing activity was seen with IFN-α. This suggests that the activity in CM is not IL-6 or OSM. Experiments with anti-gp130 antibody would confirm that activity is due to a member of this family.

It is possible that activity is due to a combination of factors. OSM, LIF, and/or another member of the IL-6 group, combined with IFN-α or IFN-β could generate AP-inducing activity in A549 cells equivalent to that of CM, but would not account for the induction of AP in H441 cells. A combination of optimal concentrations of IFN-α, IL-6 and insulin were shown previously to have equivalent activity to conditioned medium (McCormick et al, 1995), but this would still show inhibition with anti-IL-6, subject to the reservations above. Keratinocyte growth factor (KGF, FGF-7) has been shown to induce alveolar type II cell maturation (Shiratori et al, 1996; Chelly et al, 1999) but as it had no activity in AP induction with A549 (data not shown) it was not included in this series. It could, however, have an important role in surfactant induction, if not in A549 cells, perhaps in H441.

Induction of SPA expression confirmed the differentiating activity of CM+DX in H441 cells and suggested that, unlike AP, SPA is induced by OSM, IL-6 insulin and IFN in the presence of DX. IF was also active, though less so, in the absence of DX. This was distinct from the response of A549 cells, where all conditions, other than DX alone, decreased SPA expression, though less so in the presence of DX. It would appear that the regulation of AP and SPA may be different and that A549 clearly responds differently from H441 and may be progressing along a different lineage. Induction of AP suggests that this is not towards a type I pneumocyte, but it may be towards a Clara cell or other secretory phenotype. As Clara cells also make SPA (Kalina et al, 1992) the second may be more likely. Alternatively, inappropriate type II

 *© 2000 Cancer Research Campaign*

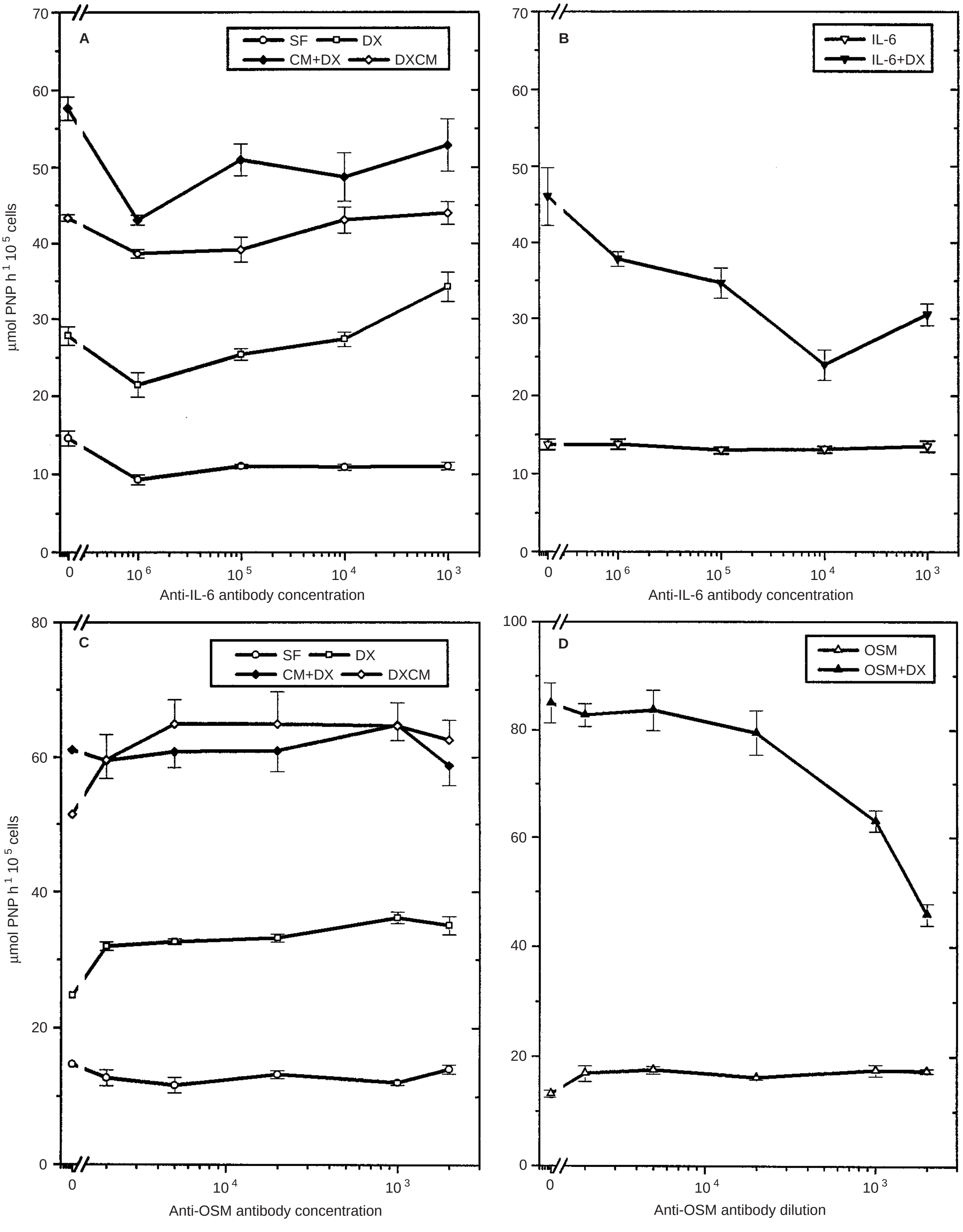

**Figure 7** Effect of disabling antibodies to OSM and IL-6 on the induction of AP by A549 cells. Conditioned medium, or recombinant human cytokine, were pretreated with antibody as described in Methods and then incubated with A549 cells as previously, and AP assayed. (**A**) Media pre-treated with anti-IL-6 antibody, (**B**) rhIL-6 pre-treated with anti-IL-6 antibody, (**C**) Media pre-treated with anti-OSM antibody, (**D**) rhOSM pretreated with anti-OSM antibody. SF, serum-free F10:DMEM; DX, F10:DMEM with 0.25 μM dexamethasone; CM+DX, fibroblast conditioned-medium with 0.25 μM DX; DXCM, medium conditioned by fibroblasts in the presence of 0.25 μM DX. Means ± s.e.m

© 2000 Cancer Research Campaign

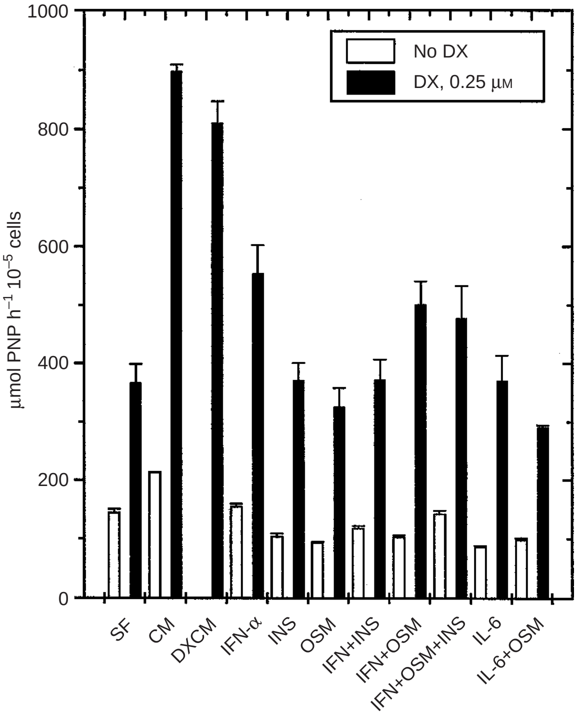

**Figure 8** Effect of fibroblast conditioned-medium (CM) and cytokines on AP activity in NCI-H441 cells. Mid-log phase H441 cells were incubated in microtitration plates in serum-free medium for 3 days with or without 0.25 μM DX, 50% CM, 10 ng ml$^{-1}$ OSM, 2.5 μg ml$^{-1}$ IL-6, 2.5 μg ml$^{-1}$ insulin and 20 ng ml$^{-1}$ IF-α, and assayed for AP as described in the Methods. Means ± s.e.m

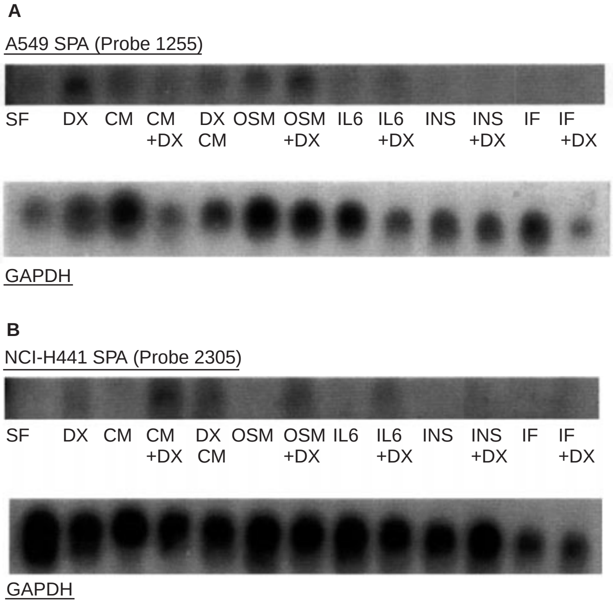

**Figure 9** Effect of fibroblast conditioned-medium (CM) and cytokines on expression of SPA mRNA in A549 and NCI-H441 cells. Radioactive cDNA was prepared from Probe 155 or 2305, specific for SPA mRNA, and hybridized to RNA extracted from A549 or NCI-H441 cells after treatment for 3 days with 50% CM, 10 ng ml$^{-1}$ OSM, 2.5 μg ml$^{-1}$ IL-6, 2.5 μg ml$^{-1}$ insulin, or 20 ng ml$^{-1}$ IF-α, in the presence and absence of 0.25 μM DX

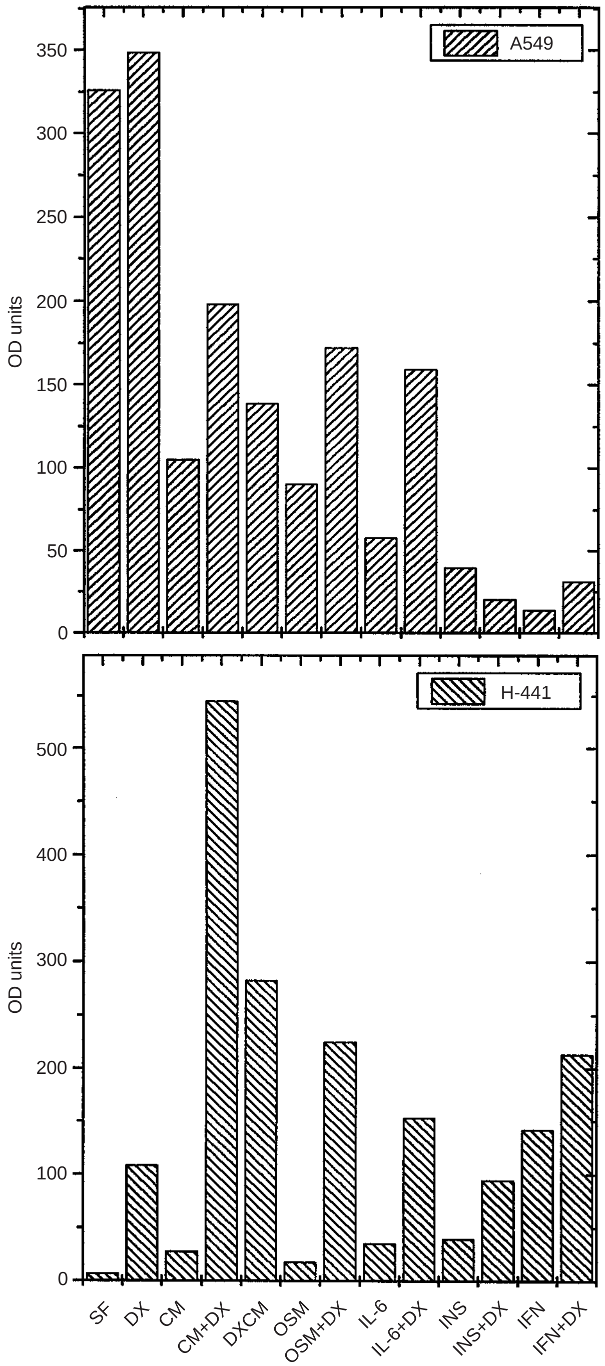

**Figure 10** Densitometric scans from autoradiographs in Figure 9. The ODs in each lane were normaliZed to the GAPDH values. OD units are nominal. Relative values can be compared within one, but not between two experiments differentiation by A549 may be due to a greater sensitivity (than H441) to the absence of the correct extracellular matrix constituents required for type II maturation (Fraslon et al, 1993; Griffin et al, 1993). Although different probes were used for the two cell lines, results were similar for both probes with SF, CM, DX, CM+DX and DXCM in A549 (cytokines not tested; data not shown), but the possibility remains that apparent differences from

 *© 2000 Cancer Research Campaign*

H441 were due to different hybridization characteristics of the two probes or differences in the regulation of expression of mRNA for SP-A1 and SP-A2 in the two cell lines (Scavo et al, 1998).

In conclusion, IL-6 and OSM are among the most active cytokines in the induction of AP activity in A549 cells, but the lack of inhibition of OSM and IL-6 by blocking antibodies, and their lack of AP-inducing activity with H441 cells, suggests that the active paracrine factor(s) produced by lung fibroblasts have not yet been identified. Purification or cloning of the factor or factors present in conditioned medium, plus the recreation of the correct microenvironment with extracellular matrix constituents such as laminin and proteoglycans, and the presence of cells or conditioned medium from primary cultures from the type II lineage, may throw further light on this.

### ACKNOWLEDGEMENTS

We are grateful to the Cancer Research Campaign for their support. We are also grateful to Riken for donation of molecular probes for surfactant proteins. Particular thanks are due to Ms Lynn McGarry for expert help and advice on densitometric quantitation of autoradiographs from Northern blots.

### REFERENCES

Akira S (1997) IL-6-regulated transcription factors. *Int J Biochem Cell Biol* **29**: 1401–1418

Alcorn JL, Smith ME, Smith JF, Margraf LR and Mendelson CR (1997) Primary cell culture of human type II pneumonocytes: maintenance of a differentiated phenotype and transfection with recombinant adenoviruses. *Am J Respir Cell Mol Biol* **17**: 672–682

Beers MF, Solarin KO, Guttentag SH, Rosenbloom J, Kormilli A, Gonzales LW and Ballard PL (1998) TGF-beta1 inhibits surfactant component expression and epithelial cell maturation in cultured human fetal lung. *Am J Physiol* **275**: L950–960

Boeuf H, Hauss C, Graeve FD, Baran N and Kedinger C (1997) Leukemia inhibitory factor-dependent transcriptional activation in embryonic stem cells. *J Cell Biol* **138**: 1207–1217

Bruce AG, Linsley PS and Rose TM (1992) Oncostatin M. *Prog. Growth Factor Res* 4: 157–170

Cai J, Zheng T, Lotz M, Zhang Y, Masood R and Gill P (1997) Glucocorticoids induce Kaposi's sarcoma cell proliferation through the regulation of transforming growth factor-beta. *Blood* **89**: 1491–1500

Chelly N, Mouhieddine-Gueddiche OB, Barlier-Mur AM, Chailley-Heu B and Bourbon JR (1999) Keratinocyte growth factor enhances maturation of fetal rat lung type II cells. *Am J Respir Cell Mol Biol* **20**: 423–432

Chen TR (1977) In situ detection of mycoplasma contamination in cell cultures by fluorescent Hoescht 33258 stain. *Exp Cell Res* **104**: 255–262

Cunha GR, Chung LWK, Shannon JM, Taguchi O and Fujii H (1983) Hormone-induced morphogenesis and growth: role of mesenchymal-epithelial interactions. *Recent Prog Horm Res* **39**: 559–598

Drach J, Lopez-Berstein G, McQueen T, Andreeff M and Mehta K (1993) Induction of differentiation in myeloid leukemia cell lines and acute promyelocytic leukemia cells by liposomal all-trans-retinoic acid. *Cancer Res* **53**: 2100–2104

Drach J, Lopez-Berstein G, McQueen T, Andreeff M and Mehta K (1993) Induction of differentiation in myeloid leukemia cell lines and acute promyelocytic leukemia cells by liposomal all-trans-retinoic acid. *Cancer Res* **53**: 2100–2104

Fraslon C, Lacaze-Masmonteil T, Zupan V, Chailley-Heu B and Bourbon JR (1993) Fetal rat lung type II cell differentiation in serum-free isolated cell culture: modulation and inhibition. *Am J Physiol* **264**: L504–516

Freshney RI (1985) Induction of differentiation in neoplastic cells. *Anticancer Res* **5**: 111–130

Freshney RI (2000) *Culture of Animal Cells, a Manual of Basic Technique*, pp. 331–332. Wiley-Liss: New York

Gearing DP, Comeau MR, Friend DJ, Gimpel SD, Thrut CJ, McGrourty J, Brasher KK, King JA, Gillis S, Mosley B, Zieger SF and Cosman D (1992) The IL-6 signal transducer, gp130: An oncostatin receptor and affinity converter for the LIF receptor. *Science* **26**: 1434–1437

Griffin M, Bhandari R, Hamilton G, Chan YC and Powell JT (1993) Alveolar type II cell–fibroblast interactions, synthesis and secretion of surfactant and type I collagen. *J Cell Sci* **105**: 423–432

Haselmann J and Goppelt-Struebe M (1997) Glucocorticoids inhibit oncostatin M-induced phospholipase A2 gene expression in human hepatoma cells. *Cytokine* **9**: 199–205

Jaskoll T, Choy HA and Melnick M (1996) The glucocorticoid-glucocorticoid receptor signal transduction pathway, transforming growth factor-beta, and embryonic mouse lung development in vivo. *Pediatric Res* 39: 749–759

Kalina M, Mason RJ and Shannon JM (1992) Surfactant protein C is expressed in alveolar type II cells but not in Clara cells of rat lung. *Am J Respir Cell Mol Biol* **6**: 594–600

Katayama N and Ogawa M (1994) Assay for murine blast cell colonies. In: *Culture of Hematopoietic Cells*, Freshney RI, Pragnell IB and Freshney MG (eds.), pp. 43–53. Wiley-Liss: New York

Kedinger M, Simon-Assmann P, Alexandre E and Haffen K (1987) Importance of a fibroblastic support for in vitro differentiation of intestinal endodermal cells and for their response to glucocorticoids. *Cell Differ* **20**: 171–182

Lillien LE, Sendtner M and Raff MC (1990) Extracellular matrix-associated molecules collaborate with ciliary neurotrophic factor to induce type-2 astrocyte development. *J Cell Biol* **111**: 635–644

Liu S and Mautone AJ (1996) Whole cell potassium currents in fetal rat alveolar type II cells cultured on Matrigel matrix. *Am J Physiol* 270: L577–586

McCormick C, Freshney RI and Speirs V (1995) Activity of interferon α, interleukin 6 and insulin in the regulation of differentiation in A549 alveolar carcinoma cells. *Br J Cancer* **71**: 232–239

Mackie AE, Freshney RI, Akturk F and Hunt G (1988) Glucocorticoids and the cell surface of human glioma cells: Relationship to cytostasis. *Br J Cancer* **58**: 101–107

Marks PA, Richon VM and Rifkind RA (1996) Cell cycle regulatory proteins are targets for induced differentiation of transformed cells: Molecular and clinical studies employing hybrid polar compounds. *Int J Hematol* **63**: 1–17

Nielsen HC, Kellogg CK and Doyle CA (1992) Development of fibroblast-type-II cell communications in fetal rabbit lung organ culture. *Biochim Biophys Acta* **1175**: 95–99

Niwa H, Burdon T, Chambers I and Smith A (1998) Self-renewal of pluripotent embryonic stem cells is mediated via activation of STAT3. *Genes Dev* **12**: 2048–2060

Piquet-Pellorce C, Grey L, Mereau A and Heath JK (1994) Are LIF and related cytokines functionally equivalent? *Exp Cell Res* **213**: 340–347

Planz B, Wang Q, Kirley SD, Lin CW and McDougal WS (1998) Androgen responsiveness of stromal cells of the human prostate: regulation of cell proliferation and keratinocyte growth factor by androgen. *J Urol* **160**: 1850–1855

Post M, Floros J and Smith BT (1984) Inhibition of lung maturation by monoclonal antibodies against fibroblast–pneumocyte factor. *Nature* **308**: 284–286

Reichardt HM and Schutz G (1998) Glucocorticoid signalling-multiple variations of a common theme. *Mol Cell Endocrinol* **146**: 1–6

Rose TM and Bruce AG (1991) Oncostatin M is a member of a cytokine family that includes leukaemia-inhibitory factor, granulocyte colony-stimulating factor and interleukin 6. *Proc Natl Acad Sci USA* **88**: 8645–8645

Sambrook J, Fritsch EF and Maniatis T (1989) *Molecular Cloning: a Laboratory Manual*. Cold Spring Harbor Laboratory Press: Cold Spring Harbor

Scavo LM, Ertsey R and Gao BQ (1998) Human surfactant proteins A1 and A2 are differentially regulated during development and by soluble factors. *Am J Physiol* **275**: L653–669

Schlessinger J, Lax I and Lemmon M (1995) Regulation of growth factor activation by proteoglycans: what is the role of the low affinity receptors? *Cell* **83**: 357–360

Shiratori M, Oshika E, Ung LP, Singh G, Shinozuka H, Warburton D, Michalopoulos G and Katyal SL (1996) Keratinocyte growth factor and embryonic rat lung morphogenesis. *Am J Respir Cell Mol Biol* **15**: 328–338

Simon-Assmann P, Kedinger M and Haffen K (1986) Immunocytochemical localization of extracellular matrix proteins in relation to rat intestinal morphogenesis. *Differentiation* **32**: 59–66

Smith AG, Heath JK, Donaldson DD, Wong GG, Moreau J, Stahl M and Rodgers D (1988) Inhibition of pluripotential stem cell differentiation by purified polypeptides. *Nature* **336**: 688–690

Speirs V, Ray KP and Freshney RI (1991) Paracrine control of differentiation in the alveolar carcinoma, A549, by human foetal lung fibroblasts. *Br J Cancer* **64**: 693–699

Taga T and Kishimoto T (1997) Gp130 and the interleukin-6 family of cytokines. *Ann Rev Immunol* **15**: 797–819

© 2000 Cancer Research Campaign 

Taga T, Narazaki M, Yasukawa K, Saito T, Miki D, Hamaguchi M, Davis S, Shoyab M, Yancopoulos GD and Kishimoto T (1992) Functional inhibition of hematopoietic and neurotrophic cytokines by blocking the interleukin 6 signal transducer gp 130. *Proc Natl Acad Sci* **89**: 10998–11001

Taimi M, Chen ZX and Breitman TR (1998) Potentiation of retinoic acid-induced differentiation of human acute promyelocytic leukemia NB4 cells by butyric acid, tributyrin, and hexamethylene bisacetamide. *Oncol Res* **10**: 75–84

Takeda T, Kurachi H, Yamamoto T, Nishio Y, Nakatsuji Y, Morishige KI, Miyake A and Murata Y (1998) Crosstalk between the interleukin-6 (IL-6)-JAK-STAT and the glucocorticoid-nuclear receptor pathway: synergistic activation of IL-6 response element by IL-6 and glucocorticoid. *J Endocrinol* **159**: 323–330

Tian L, Philp JA and Shipston MJ (1999) Glucocorticoid block of protein kinase C signalling in mouse pituitary corticotroph AtT20 D16:16 cells. *J Physiol* **516**: 757–768

Torday JS and Kourembanas S (1990) Fetal rat lung fibroblasts produce a TGF-β homologue that blocks type II cell maturation. *Dev Biol* **13**: 35–41

Wang J, Kuliszewski M, Yee W, Sedlackova L, Xu J, Tseu I and Post M (1995) Cloning and expression of glucocorticoid-induced genes in fetal rat lung fibroblasts. Transforming growth factor-beta 3. *J Biol Chem* **270**: 2722–2728

Wang J, Zhu Z, Nolfo R and Elias JA (1999). Dexamethasone regulation of lung epithelial cell and fibroblast interleukin-11 production. *Am J Physiol* **276**: L175–185

Whitsett JA, Budden A, Hull WM, Clark JC and O'Reilly, MA (1992) Transforming growth factor-beta inhibits surfactant protein A expression in vitro. *Biochim Biophys Acta* **1123**: 257–262

Wiegers GJ and Reul JM (1998) Induction of cytokine receptors by glucocorticoids: functional and pathological significance. *Tr Pharmacol Sci* **19**: 317–321

Yan G, Fukabori Y, Nikolaropoulost S, Wang F and McKeehan WL (1992) Heparin binding keratinocyte growth factor is a candidate stromal to epithelial cell andromedin. *Mol Endocrinol* **6**: 2123–2128

Yevdokimova N and Freshney RI (1997) Activation of paracrine growth factors by heparan sulphate induced by glucocorticoid in A549 lung carcinoma cells. *Br J Cancer* **76**: 261–289

*© 2000 Cancer Research Campaign*

## References

[PDF_00784] Akira S. (1997). IL-6-regulated transcription factors.. Int J Biochem Cell Biol.

[PDF_00786] Alcorn J. L., Smith M. E., Smith J. F., Margraf L. R., Mendelson C. R. (1997). Primary cell culture of human type II pneumonocytes: maintenance of a differentiated phenotype and transfection with recombinant adenoviruses.. Am J Respir Cell Mol Biol.

[PDF_00790] Beers M. F., Solarin K. O., Guttentag S. H., Rosenbloom J., Kormilli A., Gonzales L. W., Ballard P. L. (1998). TGF-beta1 inhibits surfactant component expression and epithelial cell maturation in cultured human fetal lung.. Am J Physiol.

[PDF_00794] Boeuf H., Hauss C., Graeve F. D., Baran N., Kedinger C. (1997). Leukemia inhibitory factor-dependent transcriptional activation in embryonic stem cells.. J Cell Biol.

[PDF_00797] Bruce A. G., Linsley P. S., Rose T. M. (1992). Oncostatin M.. Prog Growth Factor Res.

[PDF_00799] Cai J., Zheng T., Lotz M., Zhang Y., Masood R., Gill P. (1997). Glucocorticoids induce Kaposi's sarcoma cell proliferation through the regulation of transforming growth factor-beta.. Blood.

[PDF_00802] Chelly N., Mouhieddine-Gueddiche O. B., Barlier-Mur A. M., Chailley-Heu B., Bourbon J. R. (1999). Keratinocyte growth factor enhances maturation of fetal rat lung type II cells.. Am J Respir Cell Mol Biol.

[PDF_00805] Chen T. R. (1977). In situ detection of mycoplasma contamination in cell cultures by fluorescent Hoechst 33258 stain.. Exp Cell Res.

[PDF_00807] Cunha G. R., Chung L. W., Shannon J. M., Taguchi O., Fujii H. (1983). Hormone-induced morphogenesis and growth: role of mesenchymal-epithelial interactions.. Recent Prog Horm Res.

[PDF_00810] Drach J., Lopez-Berestein G., McQueen T., Andreeff M., Mehta K. (1993). Induction of differentiation in myeloid leukemia cell lines and acute promyelocytic leukemia cells by liposomal all-trans-retinoic acid.. Cancer Res.

[PDF_00816] Fraslon C., Lacaze-Masmonteil T., Zupan V., Chailley-Heu B., Bourbon J. R. (1993). Fetal rat lung type II cell differentiation in serum-free isolated cell culture: modulation and inhibition.. Am J Physiol.

[PDF_00819] Freshney R. I. (1985). Induction of differentiation in neoplastic cells.. Anticancer Res.

[PDF_00823] Gearing D. P., Comeau M. R., Friend D. J., Gimpel S. D., Thut C. J., McGourty J., Brasher K. K., King J. A., Gillis S., Mosley B. (1992). The IL-6 signal transducer, gp130: an oncostatin M receptor and affinity converter for the LIF receptor.. Science.

[PDF_00827] Griffin M., Bhandari R., Hamilton G., Chan Y. C., Powell J. T. (1993). Alveolar type II cell-fibroblast interactions, synthesis and secretion of surfactant and type I collagen.. J Cell Sci.

[PDF_00830] Haselmann J., Goppelt-Struebe M. (1997). Glucocorticoids inhibit oncostatin M-induced phospholipase A2 gene expression in human hepatoma cells.. Cytokine.

[PDF_00833] Jaskoll T., Choy H. A., Melnick M. (1996). The glucocorticoid-glucocorticoid receptor signal transduction pathway, transforming growth factor-beta, and embryonic mouse lung development in vivo.. Pediatr Res.

[PDF_00836] Kalina M., Mason R. J., Shannon J. M. (1992). Surfactant protein C is expressed in alveolar type II cells but not in Clara cells of rat lung.. Am J Respir Cell Mol Biol.

[PDF_00842] Kédinger M., Simon-Assmann P., Alexandre E., Haffen K. (1987). Importance of a fibroblastic support for in vitro differentiation of intestinal endodermal cells and for their response to glucocorticoids.. Cell Differ.

[PDF_00845] Lillien L. E., Sendtner M., Raff M. C. (1990). Extracellular matrix-associated molecules collaborate with ciliary neurotrophic factor to induce type-2 astrocyte development.. J Cell Biol.

[PDF_00848] Liu S., Mautone A. J. (1996). Whole cell potassium currents in fetal rat alveolar type II cells cultured on Matrigel matrix.. Am J Physiol.

[PDF_00853] Mackie A. E., Freshney R. I., Akturk F., Hunt G. (1988). Glucocorticoids and the cell surface of human glioma cells: relationship to cytostasis.. Br J Cancer Suppl.

[PDF_00856] Marks P. A., Richon V. M., Rifkind R. A. (1996). Cell cycle regulatory proteins are targets for induced differentiation of transformed cells: Molecular and clinical studies employing hybrid polar compounds.. Int J Hematol.

[PDF_00850] McCormick C., Freshney R. I., Speirs V. (1995). Activity of interferon alpha, interleukin 6 and insulin in the regulation of differentiation in A549 alveolar carcinoma cells.. Br J Cancer.

[PDF_00859] Nielsen H. C., Kellogg C. K., Doyle C. A. (1992). Development of fibroblast-type-II cell communications in fetal rabbit lung organ culture.. Biochim Biophys Acta.

[PDF_00862] Niwa H., Burdon T., Chambers I., Smith A. (1998). Self-renewal of pluripotent embryonic stem cells is mediated via activation of STAT3.. Genes Dev.

[PDF_00865] Piquet-Pellorce C., Grey L., Mereau A., Heath J. K. (1994). Are LIF and related cytokines functionally equivalent?. Exp Cell Res.

[PDF_00867] Planz B., Wang Q., Kirley S. D., Lin C. W., McDougal W. S. (1998). Androgen responsiveness of stromal cells of the human prostate: regulation of cell proliferation and keratinocyte growth factor by androgen.. J Urol.

[PDF_00871] Post M., Floros J., Smith B. T. (1984). Inhibition of lung maturation by monoclonal antibodies against fibroblast-pneumonocyte factor.. Nature.

[PDF_00874] Reichardt H. M., Schütz G. (1998). Glucocorticoid signalling--multiple variations of a common theme.. Mol Cell Endocrinol.

[PDF_00876] Rose T. M., Bruce A. G. (1991). Oncostatin M is a member of a cytokine family that includes leukemia-inhibitory factor, granulocyte colony-stimulating factor, and interleukin 6.. Proc Natl Acad Sci U S A.

[PDF_00881] Scavo L. M., Ertsey R., Gao B. Q. (1998). Human surfactant proteins A1 and A2 are differentially regulated during development and by soluble factors.. Am J Physiol.

[PDF_00884] Schlessinger J., Lax I., Lemmon M. (1995). Regulation of growth factor activation by proteoglycans: what is the role of the low affinity receptors?. Cell.

[PDF_00887] Shiratori M., Oshika E., Ung L. P., Singh G., Shinozuka H., Warburton D., Michalopoulos G., Katyal S. L. (1996). Keratinocyte growth factor and embryonic rat lung morphogenesis.. Am J Respir Cell Mol Biol.

[PDF_00890] Simon-Assmann P., Kedinger M., Haffen K. (1986). Immunocytochemical localization of extracellular-matrix proteins in relation to rat intestinal morphogenesis.. Differentiation.

[PDF_00893] Smith A. G., Heath J. K., Donaldson D. D., Wong G. G., Moreau J., Stahl M., Rogers D. (1988). Inhibition of pluripotential embryonic stem cell differentiation by purified polypeptides.. Nature.

[PDF_00896] Speirs V., Ray K. P., Freshney R. I. (1991). Paracrine control of differentiation in the alveolar carcinoma, A549, by human foetal lung fibroblasts.. Br J Cancer.

[PDF_00899] Taga T., Kishimoto T. (1997). Gp130 and the interleukin-6 family of cytokines.. Annu Rev Immunol.

[PDF_00903] Taga T., Narazaki M., Yasukawa K., Saito T., Miki D., Hamaguchi M., Davis S., Shoyab M., Yancopoulos G. D., Kishimoto T. (1992). Functional inhibition of hematopoietic and neurotrophic cytokines by blocking the interleukin 6 signal transducer gp130.. Proc Natl Acad Sci U S A.

[PDF_00907] Taimi M., Chen Z. X., Breitman T. R. (1998). Potentiation of retinoic acid-induced differentiation of human acute promyelocytic leukemia NB4 cells by butyric acid, tributyrin, and hexamethylene bisacetamide.. Oncol Res.

[PDF_00910] Takeda T., Kurachi H., Yamamoto T., Nishio Y., Nakatsuji Y., Morishige K. i., Miyake A., Murata Y. (1998). Crosstalk between the interleukin-6 (IL-6)-JAK-STAT and the glucocorticoid-nuclear receptor pathway: synergistic activation of IL-6 response element by IL-6 and glucocorticoid.. J Endocrinol.

[PDF_00914] Tian L., Philp J. A., Shipston M. J. (1999). Glucocorticoid block of protein kinase C signalling in mouse pituitary corticotroph AtT20 D16:16 cells.. J Physiol.

[PDF_00917] Torday J. S., Kourembanas S. (1990). Fetal rat lung fibroblasts produce a TGF beta homolog that blocks alveolar type II cell maturation.. Dev Biol.

[PDF_00919] Wang J., Kuliszewski M., Yee W., Sedlackova L., Xu J., Tseu I., Post M. (1995). Cloning and expression of glucocorticoid-induced genes in fetal rat lung fibroblasts. Transforming growth factor-beta 3.. J Biol Chem.

[PDF_00922] Wang J., Zhu Z., Nolfo R., Elias J. A. (1999). Dexamethasone regulation of lung epithelial cell and fibroblast interleukin-11 production.. Am J Physiol.

[PDF_00924] Whitsett J. A., Budden A., Hull W. M., Clark J. C., O'Reilly M. A. (1992). Transforming growth factor-beta inhibits surfactant protein A expression in vitro.. Biochim Biophys Acta.

[PDF_00927] Wiegers G. J., Reul J. M. (1998). Induction of cytokine receptors by glucocorticoids: functional and pathological significance.. Trends Pharmacol Sci.

[PDF_00929] Yan G., Fukabori Y., Nikolaropoulos S., Wang F., McKeehan W. L. (1992). Heparin-binding keratinocyte growth factor is a candidate stromal-to-epithelial-cell andromedin.. Mol Endocrinol.

[PDF_00932] Yevdokimova N., Freshney R. I. (1997). Activation of paracrine growth factors by heparan sulphate induced by glucocorticoid in A549 lung carcinoma cells.. Br J Cancer.

